# Presowing Management as a Factor Influencing Vegetative, Reproductive, and Biochemical Performance in Soybean Seed Production

**DOI:** 10.1021/acsomega.6c07818

**Published:** 2026-10-15

**Authors:** Tassila Aparecida do Nascimento de Araújo, Andreia Almeida da Silva, Eduardo Pradi Vendruscolo, Natália Alves Nogueira, Angelita Celente Martins, Flávio Ferreira da Silva Binotti

**Affiliations:** † Federal University of Pelotas, Capão do Leão, Rio Grande do Sul 96160-000, Brazil; ‡ State University of Mato Grosso do Sul, Cassilândia 79540-000, Brazil

## Abstract

Although chemical, biological, and vitamin-based seed treatments are individually well documented in soybean, comparative evidence on their combined application within a single experimental framework remains scarce. This study evaluated the effects of treating soybean seeds with a chemical product, *Azospirillum spp*. (2 mL kg^–1^ of seed), and the B-complex vitamins pyridoxine (B6) and nicotinamide (B3) (100 mg kg^–1^ of seed), applied alone and in combination, on gas exchange, vegetative growth, reproductive performance, and seed biochemical composition of soybean grown under greenhouse conditions. Seed treatments altered intercellular CO_2_ concentration and increased carboxylation efficiency, without significantly affecting net photosynthesis, stomatal conductance, transpiration, or water use efficiency. Treated plants showed greater plant height, branching, and pod number, together with higher seed number and mass, with *Azospirillum spp* producing the most consistent gains. Protein, lipid, amino acid, and soluble sugar contents increased with seed treatment, while proline remained unchanged, indicating a beneficial physiological stimulus rather than a stress response. These findings show that combining chemical, biological, and vitamin-based seed treatments improves vigor and early performance in soybean seed production and provide a practical basis for developing multi-input seed treatment protocols, although validation under field conditions is still required.

## Graphical abstract

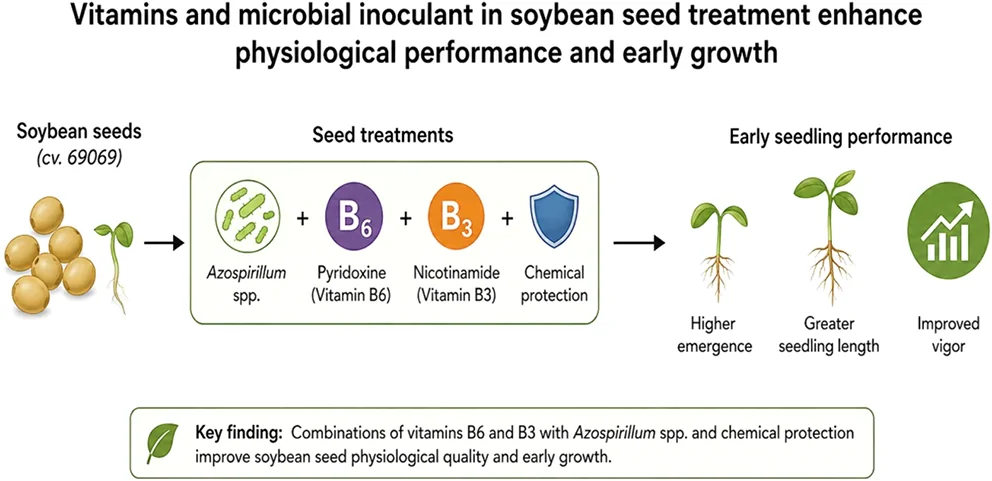

## Introduction

The cultivation of *Glycine max* (L.) Merrill stands out as one of the leading agricultural commodities on a global scale, being grown in approximately 60 countries. In Brazil, it covers about 47.40 million hectares, establishing itself as the leading export product.^1^ Its economic and nutritional importance is linked to its high oil and protein yield, widely used in human and animal feed, as well as its application in agribusiness.^2–4^ However, crop productivity has been negatively affected by climate change, which intensifies the occurrence of environmental stresses and compromises crop performance.^5^

Soybean production is frequently affected by abiotic and biotic stresses, such as water deficit, salinity, extreme temperatures, nutritional deficiencies, plant pathogens, and pests.^6^ In this context, it is essential to develop strategies that promote greater plant tolerance to these adverse conditions, ensuring the sustainability of production systems. At the same time, growing food demand has driven the intensification of agricultural production, with a focus on increasing productivity without expanding cultivated areas.^7^

Seed quality is one of the key factors determining initial plant establishment and crop success. In this regard, understanding the physiological and biochemical mechanisms involved in seed formation and development is essential. Compounds such as proteins, amino acids, and organic acids play an essential role in the formation of reserves and embryonic development, with storage proteins being particularly important because they provide amino acids during germination and the initial growth of seedlings.^8,9^

In addition, the heavy reliance on chemical inputs in agriculture has raised concerns about environmental impacts and the sustainability of production systems. Consequently, alternative practices, such as the use of biostimulants and biological agents, have gained prominence for promoting improvements in plant physiological performance and seed quality, while reducing environmental impacts.^10,11^ Among these compounds, B-complex vitamins such as nicotinamide (B3) and pyridoxine (B6) stand out, as they have demonstrated positive effects on the growth and development of various crops, including when applied through seed treatment.^12–14^

The exogenous application of nicotinamide has been associated with increased plant growth, cell expansion, and the accumulation of reserves, resulting in improvements in soybean yield components, even under stressful conditions.^15,16^ Similarly, pyridoxine acts to regulate physiological processes, contributing to increased levels of photosynthetic pigments and antioxidant compounds, thereby promoting tolerance to environmental stresses and enhancing plant productivity.^17,18^ Despite these advances, gaps remain regarding the standardization of application methodologies for these substances in soybean cultivation.

At the same time, the use of biological inputs, such as bacteria of the genus *Azospirillum spp*., has shown potential in promoting plant growth and improving nutrient use efficiency, especially in grasses, highlighting their applicability in sustainable agricultural systems.^19^ In addition, chemical seed treatments continue to be widely used, primarily for their role in controlling pests and diseases, as well as for improving the performance of seeds with lower vigor.^20^

Although the individual effects of chemical seed treatments, Azospirillum spp. inoculation, and B-complex vitamins on seed and plant performance have been documented separately, comparative evidence on their combined application to soybean seeds within a single experimental framework is still limited, which restricts current understanding of potential synergistic or antagonistic interactions among these three classes of inputs as they are increasingly combined in commercial seed treatment.^60–62^ In light of this gap, and given the need to integrate different strategies that promote seed quality and crop performance, the objective of this study was to evaluate the effects of treating soybean seeds with a chemical product, the biological agent Azospirillum spp., and the vitamins pyridoxine and nicotinamide, applied individually or in combination, on vegetative, reproductive, and biochemical performance, with the aim of contributing to the improvement of the crop’s physiological and productive quality.^63^ We hypothesized that combining Azospirillum spp. and B-complex vitamins with a standard chemical seed treatment would produce greater improvements in soybean vigor, growth, and seed biochemical composition than any of these inputs applied alone, reflecting complementary modes of action among the three input classes.

## Material and Methods

### Characterization of the Experimental Area

The experiment was conducted at two locations, with one of the stages carried out at the facilities of the State University of Mato Grosso do Sul (UEMS) in the municipality of Cassilândia, Mato Grosso do Sul (19°06′48″ S, 51°44′03″ W). This phase was carried out in a controlled environment, in a greenhouse measuring 18.0 × 8.0 × 4.0 m, with a total area of 144 m^2^, covered with low-density polyethylene (LDPE) film with light-diffusing properties.

The greenhouse featured a roof vent covered with white shade cloth providing 30% shading, as well as black side and front screens, also providing 30% shading, installed at a 90° angle to control incident radiation and ensure adequate ventilation of the environment. For additional thermal control, a movable aluminized heat-reflective screen (LuxiNet) was used, positioned beneath the LDPE covering.

Throughout the experimental period, indoor environmental conditions were monitored, with continuous recording of air temperature and relative humidity; the average values and variations are shown in Figure 1.

**Figure 1:**
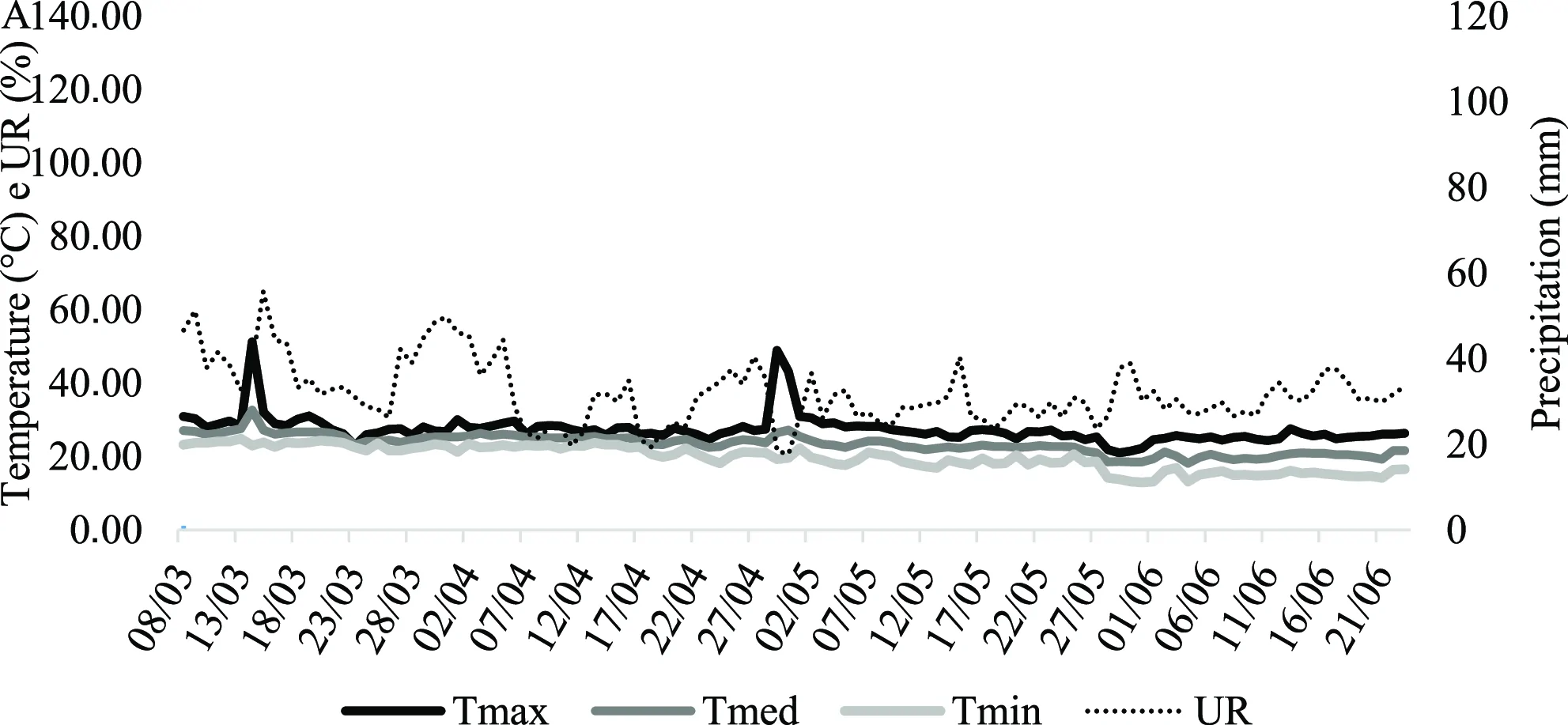
Weather conditions during soybean cultivation in a greenhouse. at the Experimental Farm of the Mato Grosso do Sul State University, Cassilândia University Unit.

The experiment was conducted using a randomized block design, with seven treatments and four replicates, for a total of 28 experimental units. Soybean seeds of the 69IX69 cultivar were used; seed treatments were applied using the following products: the chemical Vitavax Thiram 200 FS (200 g/L), the biological (*Azospirillum spp*., 2.0 mL/kg of seed), the vitamin pyridoxine (pyridoxine B6 hydrochloride, 100 mg/kg of seed), and nicotinamide (niacinamide B3 hydrochloride, 100 mg/kg of seed).

In addition, the composition of the products used in seed treatment is detailed below. The chemical used was Vitavax Thiram 200 FS, whose formulation includes the active ingredients carboxin (5,6-dihydro-2-methyl-1,4-oxathiin-3-carboxanilide) and thiram (tetramethylthiuram disulfide), both at a concentration of 200 g L^–1^ (20.0% w/v). The formulation also contains ethylene glycol at a concentration of 249 g L^–1^ (24.9% w/v) and other inert components and adjuvants totaling 507 g L^–1^ (50.7% w/v).

Functionally, this fungicide combination was adopted as the chemical baseline because carboxin is a systemic carboxamide fungicide that inhibits succinate dehydrogenase in the fungal mitochondrial respiratory chain (Complex II), providing effective control of basidiomycete pathogens such as *Rhizoctonia solani*, whereas thiram is a protective, contact dithiocarbamate fungicide with broad-spectrum activity against soil- and seed-borne fungi (e.g., *Fusarium* spp., *Aspergillus* spp., *Penicillium* spp.). Together, these active ingredients protect the seed and the emerging seedling from pathogen attack during the critical germination and early establishment phase, which is why this commercial formulation was used as the standard chemical seed treatment against which the biological and vitamin-based inputs were compared in the present study.

Biological treatment was carried out using *Azospirillum spp*. at a concentration of 2.0 mL kg^–1^ of seeds. In the vitamin treatment group, pyridoxine hydrochloride (vitamin B6) and nicotinamide hydrochloride (vitamin B3) were used, both applied at a concentration of 100 mg kg^–1^ of seeds. The vitamins were purchased from Orion Produtos e Serviços de Laboratório Ltd.a.

The seeds used in this study were provided by an agricultural company and had not undergone any prior chemical treatment. Upon receipt, they were subjected to experimental treatments, which consisted of the application of a chemical treatment or the biological agent alone, as well as combinations of these with vitamins, in order to evaluate their effects on the physiological quality of the seeds. The treatments were distributed as follows: T1 control (no application), T2 (*Azospirillum spp*.), T3 (chemical), T4 (pyridoxine + chemical), T5 (Nicotinamide + Chemical), T6 (Pyridoxine + *Azospirillum* + Chemical), and T7 (Nicotinamide + *Azospirillum* + Chemical).

The treatment structure was built around the chemical seed treatment as the baseline input, since it represents current standard commercial practice, and tested the addition of a biological agent and B-complex vitamins as complementary strategies rather than as a complete factorial design. Vitamin-only and vitamin+*Azospirillum*-only treatments, without the chemical carrier, were not included because the individual efficacy of pyridoxine and nicotinamide seed treatments has already been demonstrated by our research group and others (refs 12,15,18,39 ); the present study instead focused on how these vitamins perform when incorporated into the chemical- and biological-based protocols actually used in commercial soybean seed treatment. Pyridoxine (B6) and nicotinamide (B3) were selected among B-complex vitamins because both have shown consistent, dose-dependent effects on seedling growth and stress tolerance in legume and cereal species evaluated by our group under comparable application rates, providing a basis for comparison across crops.

Each experimental unit consisted of a black polyethylene pot with a volume of 8 L, filled with previously amended Neossolo Quartzarênico soil, adjusted to a base saturation of 70% and fertilized with the equivalent of 150 kg of P_2_O_5_ ha^–1^ and 30 kg of K_2_O ha^–1^, using micronutrient-enriched superphosphate (Yoorin, Poços de Caldas, Brazil) and potassium chloride as sources, respectively. After treatment, the seeds were spread out on trays and left for 24 h in a protected area to dry, with the aim of achieving surface drying. Sowing was then carried out by placing five seeds per pot at a depth of approximately 3 cm, followed by irrigation. After the cotyledon leaves emerged, thinning was performed, leaving two plants per experimental unit. Topdressing was applied 35 days after sowing, using the equivalent of 60 kg of K2O per hectare. Weeds were controlled manually throughout the growing season, and no need for pest or disease control was observed.

Twenty-five days after emergence, assessments were conducted of the physiological characteristics associated with the plants’ photosynthetic performance. The variables analyzed included net photosynthetic rate (A), stomatal conductance (gS), intracellular CO_2_ concentration (Ci), and transpiration rate (E). Measurements were taken in the morning, between 8:00 a.m. and 10:00 a.m., a time when plants exhibit maximum gas exchange activity, using a portable infrared gas exchange analyzer (LCi, ADC Bioscientific, Hertfordshire, United Kingdom). Instantaneous carboxylation efficiency (ICE) was estimated using the ratio of net photosynthetic rate to internal CO_2_ concentration (A/Ci), while water use efficiency (WUE) was determined by the ratio of net photosynthetic rate to transpiration rate (A/E).

At the end of the production cycle, when the plants reached physiological maturity, harvesting was carried out, followed by an assessment of productivity-related characteristics. The following were measured: stem diameter (using a caliper), plant height (using a ruler), number of branches, number of pods per plant, number of seeds per plant, pod length (using a caliper), and seed weight per plant. For these measurements, a graduated ruler was used to measure plant height, a digital caliper for stem diameter, and a semianalytical balance to determine seed weight per plant.

Following harvest and morphological and yield assessments, the biochemical compounds in the soybeans were quantified. These analyses were conducted at the Flávio Farias da Rocha Seed Analysis Teaching Laboratory of the Eliseu Maciel School of Agronomy, part of the Federal University of Pelotas (FAEM/UFPel), located in the municipality of Capão do Leão, Rio Grande do Sul. The following biochemical parameters were determined, each with four replicates per treatment:

### Extraction and Quantification of Total Proteins

Total protein extraction and quantification were performed according to the method.^21^ To this end, 0.250 g of seed meal, previously ground in a mill, was weighed and transferred to Falcon tubes. Next, 20 mL of KH_2_PO_4_ solution, adjusted to pH 6.8, was added.

The samples were shaken and then centrifuged for 15 min at 3000 rpm. Protein content was determined on the same day as the extraction. For quantification, 0.1 mL of the supernatant was transferred to test tubes, to which 0.1 mL of distilled water and 5 mL of Coomassie Brilliant Blue reagent were added. Measurements were taken using a spectrophotometer at a wavelength of 595 nm. The results were expressed in μg of protein per gram of seed.

### Determination of Total Soluble Sugars

Total soluble sugars were quantified using the method.^22^ One milliliter of each sample, as well as a blank (water) and glucose standard solutions (10 to 150 μg mL^–1^), was collected in test tubes kept on ice.

Next, 3 mL of antrone solution (0.15% w/v in concentrated sulfuric acid), which had been precooled, was added to each tube, which was immediately sealed with glass beads. After 15 min, the tubes were shaken and incubated in a water bath at 90 °C for 20 min. Subsequently, the samples were kept in the dark until they reached room temperature. After further shaking, the optical density of the standards and samples was determined using a spectrophotometer at 620 nm (Biospectro, model SP-22). The results were expressed in μg/mL.

### Amino Acid Quantification

The seedling extracts were properly diluted in ultrapure water to a final volume of 1 mL in test tubes. Next, 0.5 mL of 0.2 M citrate buffer (pH 5.0), 0.2 mL of 5% ninhydrin reagent in methylcellosolve (ethylene glycol monomethyl ether), and 1 mL of 2% (v/v) KCN in methylcellosolve, prepared from a 0.01 M aqueous KCN solution.

The tubes were shaken, sealed with glass beads, and incubated in a water bath at 100 °C for 20 min. After cooling in the dark to room temperature, 1.3 mL of 60% ethanol was added, bringing the final volume to 4 mL, followed by further shaking.

The optical density of the samples and leucine standards (0 to 200 nmol) was measured at 570 nm, with four replicates per sample. The results were expressed in μmol g^–1^ of fresh weight (FW).

### Extraction and Quantification of Total Lipids

To determine the total lipid content, the crucibles were first weighed, dried in an oven for 2 h, cooled in a desiccator, and weighed again. Next, 3 g of sample were weighed, and 16 mL of MCW solution (1:2:0.8) and 8 mL of chloroform were added to appropriate vials.

The mixture of solvents, together with distilled water, promoted phase separation. The samples were vortexed for 30 min, followed by the addition of 8 mL of 1.5% sodium sulfate, with further vortexing for 2 min. Subsequently, 8 mL of chloroform was added, and the samples were allowed to stand for phase separation.

After separation, 10 mL of the lower (chloroform) phase was collected and filtered through a funnel containing 1 g of anhydrous sodium sulfate to remove residual water. From the filtrate, 4 mL was transferred to preweighed and tared crucibles. The samples were placed in an oven at 70 °C until the solvent had completely evaporated (approximately 15 to 20 min), cooled in a desiccator, and weighed on an analytical balance. The total lipid content (%) was calculated using the following equation

$$\mathrm{Total}\;\mathrm{lipids}\;\left(\%\right)=\left(\left(\left(P_{2}‐T\right)\times 4\right)/P_{1}\right)\times 100$$

### Quantification of Proline

Prolin quantification was performed according.^23^ In test tubes, 100 μL of 0.1 M glycine, 300 μL of MCW extract, 2.6 mL of Milli-Q water, and 2 mL of concentrated acetic acid were added. After homogenization, 2 mL of the ninhydrin reagent, prepared with 600 mg of ninhydrin, 15 mL of concentrated acetic acid, and 10 mL of 6 mol L^–1^ phosphoric acid (H_3_PO_4_), were added.

The mixture was incubated in a water bath at 100 °C for 35 min, followed by rapid cooling in an ice bath. Next, 4 mL of concentrated toluene was added with vigorous stirring. The absorbance of the supernatant (toluene phase) was measured in a spectrophotometer at 515 nm. The standard curve was prepared using proline solutions at concentrations of 0; 0.5; 1; 2; 3; and 4 μg mL^–1^. The results were expressed in μmol g^–1^.

### Statistical Analysis

The data obtained were analyzed using analysis of variance (ANOVA), and the means were compared using the Scott-Knott test at a 5% significance level. Statistical analyses were performed using SISVAR software.^24^ Pearson’s correlation coefficient was also calculated (R software version 4.3.3, qgraph package) using the correlation matrix. Multivariate analysis was performed using canonical correlation analysis (Candisc package) and principal component analysis (Ggfortify and Factoextra packages) applied in R software.^24^

## Results

Figure 2 shows the significant effects of the treatments on the intercellular CO_2_ concentration (C_i_) and carboxylation efficiency (EIC_i_), demonstrating that the application of the products influenced CO_2_ diffusion into the leaf mesophyll and Rubisco’s biochemical capacity to fix carbon. The variation in C_i_ may be associated with changes in stomatal conductance or photosynthetic metabolism, while the change in EIC_i_ suggests differences in the efficiency of the Calvin-Benson cycle in response to the applied treatments.

**Figure 2:**
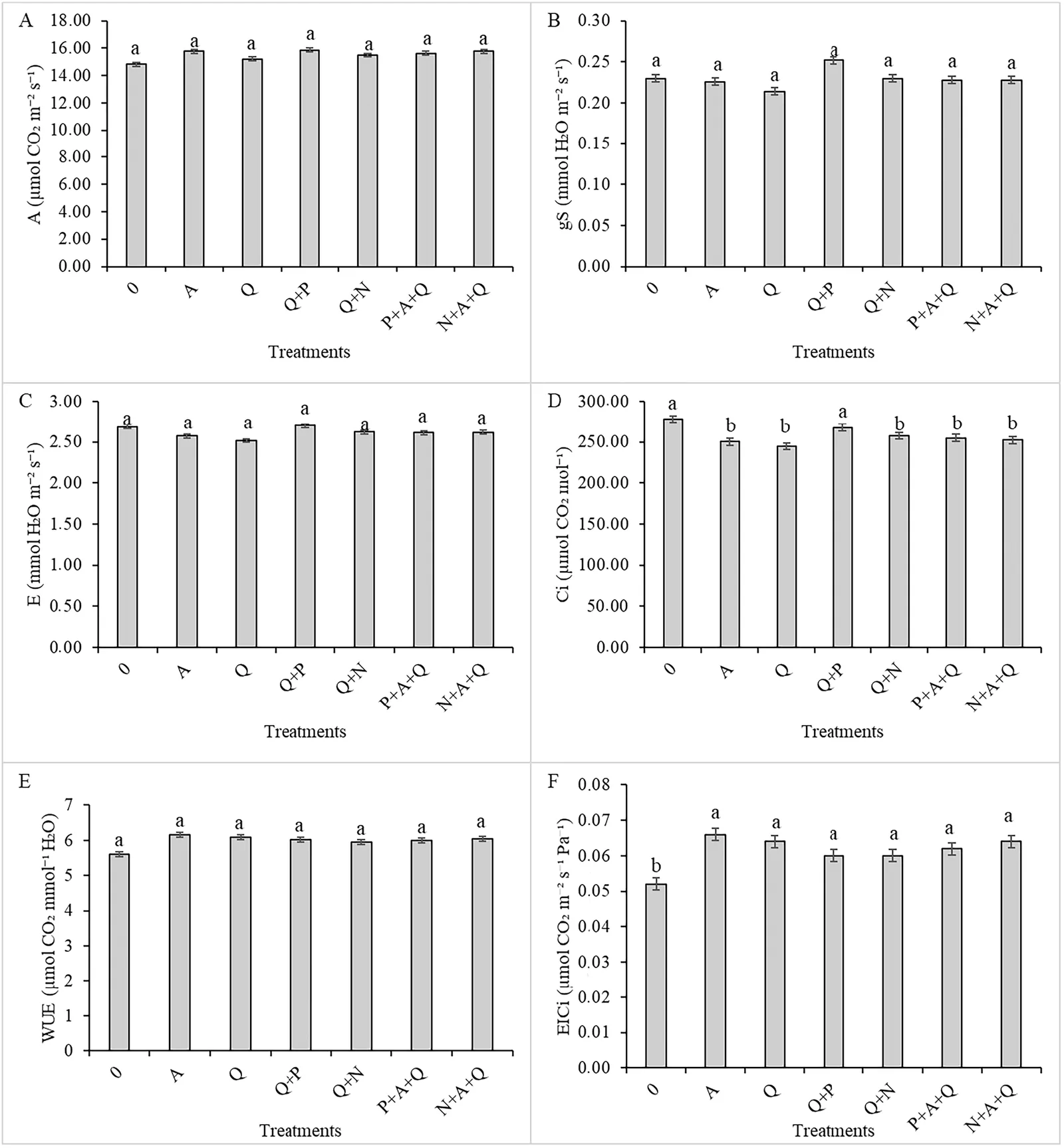
Net CO_2_ uptake (A), transpiration rate (B), stomatal conductance (C), CO_2_ concentration in the leaf mesophyll (D), water use efficiency (E), and carboxylation efficiency (F) of soybean plants under different seed treatment applications. Different letters indicate that the means belong to different groups based on the Scott-Knott test at a 5% probability level.

The net CO_2_ assimilation rate (A) did not show significant differences among the treatments, ranging from 14.5 to 16.5 μmol CO_2_ m^–2^ s^–1^ (Figure 2A). This behavior suggests that the treatments employed did not promote significant changes in carbon fixation, indicating that the crop’s photosynthetic capacity was maintained regardless of the treatment applied.

Stomatal conductance (g_s_) did not differ significantly among treatments (Figure 2B). The transpiration rate (Figure 2E) did not differ significantly among treatments, with mean values close to 2.5 mmol H_2_O m^–2^ s^–1^ (Figure 2C).

The intercellular CO_2_ concentration (C_i_) was significantly higher in untreated seeds and in the Q + P treatments, while the others showed lower values (Figure 2D). It is worth noting that the lower CO_2_ concentration in the mesophyll may be associated with increased photosynthetic fixation or stomatal restriction, reducing CO_2_ uptake. Furthermore, the relationships between stomatal conductance (g_s_), internal CO_2_ concentration, and photosynthetic performance are also related to increased C_i_, accompanied by higher g_s_. On the other hand, under stress conditions, such as water deficiency, the increase in C_i_ may be associated with nonstomatal limitations.

There were no significant differences in water use efficiency (WUE) among the treatments, with mean values close to 6.0 μmol CO_2_ mmol^–1^ H_2_O (Figure 2E). This result indicates that the ratio between CO_2_ assimilation and transpiration was maintained, suggesting that the applied treatments did not compromise the crop’s water use efficiency. Carboxylation efficiency (EIC_i_) was significantly lower in the untreated control compared to the other treatments, which did not differ from one another (Figure 2F). This result suggests that the applied treatments favored Rubisco efficiency in CO_2_ fixation, ensuring a higher assimilation rate for the same intercellular CO_2_ concentration. It is worth noting that the lower efficiency observed in the untreated treatment may be associated with biochemical limitations reducing the conversion of CO_2_ into sugars.

Taken together, the results indicate that the treatments applied altered intercellular CO_2_ concentration and carboxylation efficiency, without significant effects on net photosynthesis, stomatal conductance, transpiration, or water use efficiency, suggesting a specific effect on Rubisco carboxylation capacity rather than a broad enhancement of gas exchange.

Analysis of seedling stem diameter revealed no statistically significant differences among treatments (*p* > 0.05), as indicated by the comparison of means (Figure 3A). This lack of statistical difference suggests that this morphological trait is not very sensitive to variations in the treatment methods used. Neither the individual treatments nor the combinations of biostimulants and chemical products were sufficient to promote significant increases in stem diameter.

**Figure 3:**
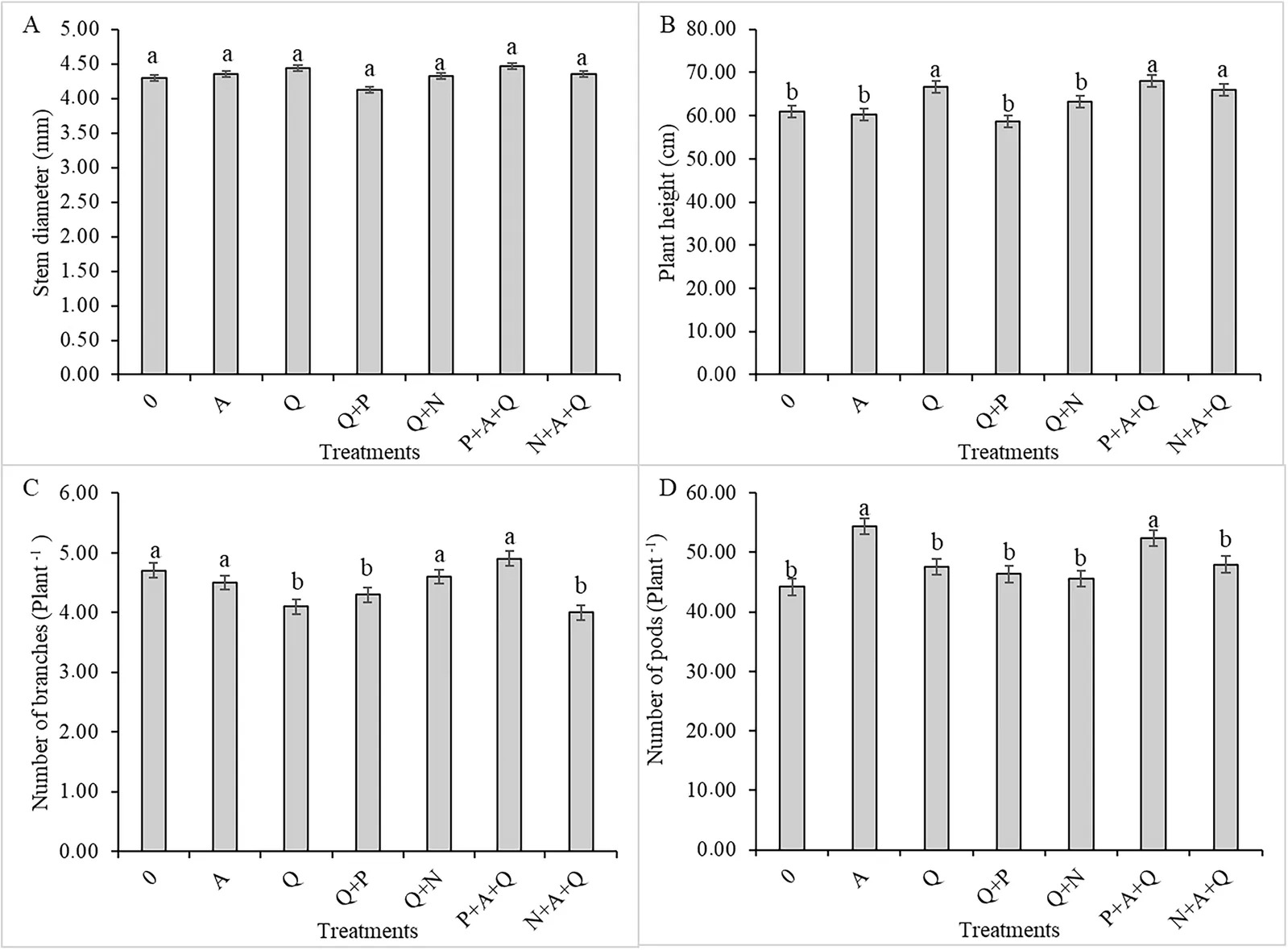
Stem diameter (A), plant height (B), number of branches per plant (C), and number of pods per treatment (D) for soybean plants under different treatments.

On the other hand, plant height was significantly influenced by the seed treatments (*p* < 0.05). Treatments T3, T6, and T7 had the highest mean heights, with averages exceeding 68 cm, differing statistically from the other treatments. In contrast, treatments T1, T2, T4, and T5 resulted in lower mean values, ranging from 62 to 64 cm. These findings indicate that the application of chemical treatments, either alone or in combination with pyridoxine, nicotinamide, and Azospirillum spp., promoted a positive response in seedling height growth (Figure 3B).

In addition, the number of branches per plant also showed significant variation among the treatments (*p* < 0.05). Treatments T1, T2, T5, and T6 had the highest mean branching values, which did not differ statistically from one another, with means exceeding 4.7 branches per plant. In contrast, treatments T3, T4, and T7 had a lower number of branches, with averages below 4.3 branches per plant. These results indicate that not all treatment combinations favored branching, and antagonistic effects may occur in certain interactions (Figure 3C).

Finally, the number of pods per plant was also significantly affected by the treatments (*p* < 0.05). Treatments T2 and T6 stood out with the highest mean values, reaching approximately 55 pods per plant, which differed statistically from the other treatments. In contrast, the control (T1), the chemical treatment alone (T3), and the combinations T4, T5, and T7 had lower means, ranging from 45 to 48 pods per plant, with no significant differences among them. These results suggest that both the isolated use of *Azospirillum spp*. and its combination with pyridoxine and chemical treatment exerted beneficial effects on pod formation (Figure 3D).

The results obtained in this study (Figure 3A, B, C, and D) show that treatments containing pyridoxine were associated with significant changes in morphological and reproductive parameters of soybean, as shown in Figures 1, 2, and 3; cellular protection and biotic-stress resistance were not directly assessed in this study and are proposed here only as plausible mechanisms warranting confirmation in future work. The results indicate that the substance had positive effects throughout different phenological stages of the crop, enhancing the overall performance of the plants.

For the number of seeds per plant, statistically significant differences were observed among the treatments (*p* < 0.05), as shown in Figure 4A. Treatment (T2) resulted in the highest mean number of seeds per plant, differing significantly from the others. On the other hand, the other treatments, including (T1), (T3), (T4), and (T5), as well as treatments (T6) and (T7), showed no statistically significant differences among themselves and resulted in a lower number of seeds per treatment when compared to T2 (Figure 4A).

**Figure 4:**
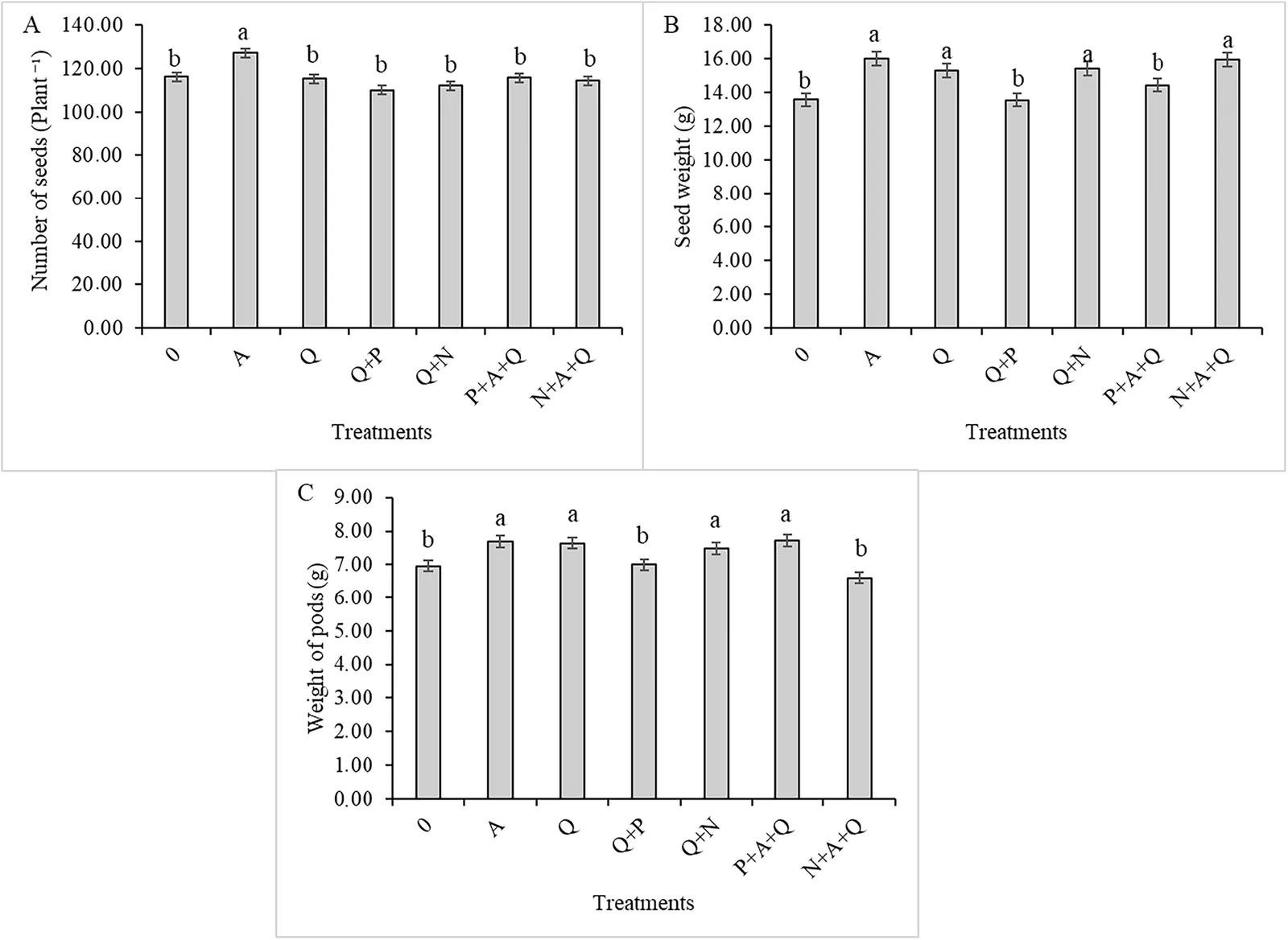
Number of seeds per treatment (A), seed weight per treatment (B), and pod weight per treatment (C) for soybean plants. Different letters indicate that the means belong to different groups based on the Scott-Knott test at the 5% significance level. Source: Prepared by the author.

Similarly, for the seed weight variable by treatment, statistically significant differences were also observed among the treatments (*p* < 0.05). In this case, treatments T2, T3, T5, and T7 had the highest mean seed mass values, constituting the statistically superior group. In contrast, treatments T1, T4, and T6 had the lowest mean values, differing from the others (Figure 4B).

In addition, the pod weight variable per treatment was significantly influenced by the seed treatments (*p* < 0.05), as shown in Figure 4C. Treatments T2, T3, T5, and T6 yielded the highest mean pod weights, ranging from approximately 7.8 to 8.2 g, and were classified in the statistically superior group. In contrast, treatments T1, T4, and T7 had the lowest mean values, with masses ranging from 7.0 to 7.3 g, differing significantly from the others.

As shown in Figure 4A, B, and C, the positive effect of the treatments on the reproductive stage of soybeans was a key factor in the increase in seed yield observed in this study. This effect is directly linked to the application of bioactive compounds, both individually and in combination, as evidenced by the formation of a consistent response group across the tested concentrations, which significantly impacted the evaluated traits.

Biostimulants composed of vitamins, biological agents, and chemical substances have gained prominence as effective strategies in modern agriculture, as they contribute to improving the physiological quality of crops and mitigating various types of stress. Among these compounds, vitamins play a significant role in cellular protection by activating antioxidant systems, which enhance plants’ ability to tolerate biotic stress conditions. Specifically, nicotinamide has been shown to have positive effects on vegetative growth, promoting cell expansion and favoring the accumulation of reserve substances, which is reflected in significant increases in productivity, as shown in Figure 4B and 4C.

At the same time, chemical compounds continue to play a key role in plant protection and are widely used to control pests and diseases, thereby ensuring that crop yields remain high. However, it has become necessary to adopt an integrated approach that combines the use of vitamin-based biostimulants, biological agents, and chemical pesticides. The treatments comprising these three classes of inputs yielded the best results in terms of plant productivity and resilience when compared to the control treatment, while also having a lower environmental impact, thereby reinforcing the principles of technically efficient and environmentally responsible agriculture.

The analysis of variance revealed a significant effect of seed treatments on the levels of protein, proline, lipids, amino acids, and total soluble sugars, as shown in Table 1. For protein, the treatment factor showed a highly significant difference, demonstrating the sensitivity of this biochemical component to the interventions evaluated. Similarly, the levels of proline, lipids, amino acids, and total soluble sugars also responded significantly to the treatments, indicating metabolic changes associated with the conditions imposed during the seed production phase.

**Table 1:** Summary of the Analysis of Variance for Protein, Proline, Lipids, Amino Acids, and Total Soluble Sugars Subjected to Different Seed Treatments^a^

QM residue						
FV	GL	Protein	Proline	Lipid	Amino acid	Total soluble sugar
Treat	6	<0.01*	0.03*	<0.01*	<0.01*	<0.01*
Rep	3	0.24	0.90	0.96	0.40	0.92
Erro	18	1.08	0.41	1.30	2.11	0.00
Media		18.95	9.01	21.06	18.88	0.31
CV (%)		5.49	7.11	5.42	7.69	17.5

a **Significant at the 1% level; *significant at the 5% level; ns: not significant. Source: Prepared by the author.

Figure 5A shows that the treatments resulted in significant differences in seed protein content. The treatment with *Azospirillum spp*. (A) showed the highest value observed, forming the statistically superior group, along with the combinations of (Q + P + A) and (Q + N + A). This result demonstrates that the presence of *Azospirillum spp*., either alone or in combination with the other inputs, leads to a substantial increase in seed protein accumulation.

**Figure 5:**
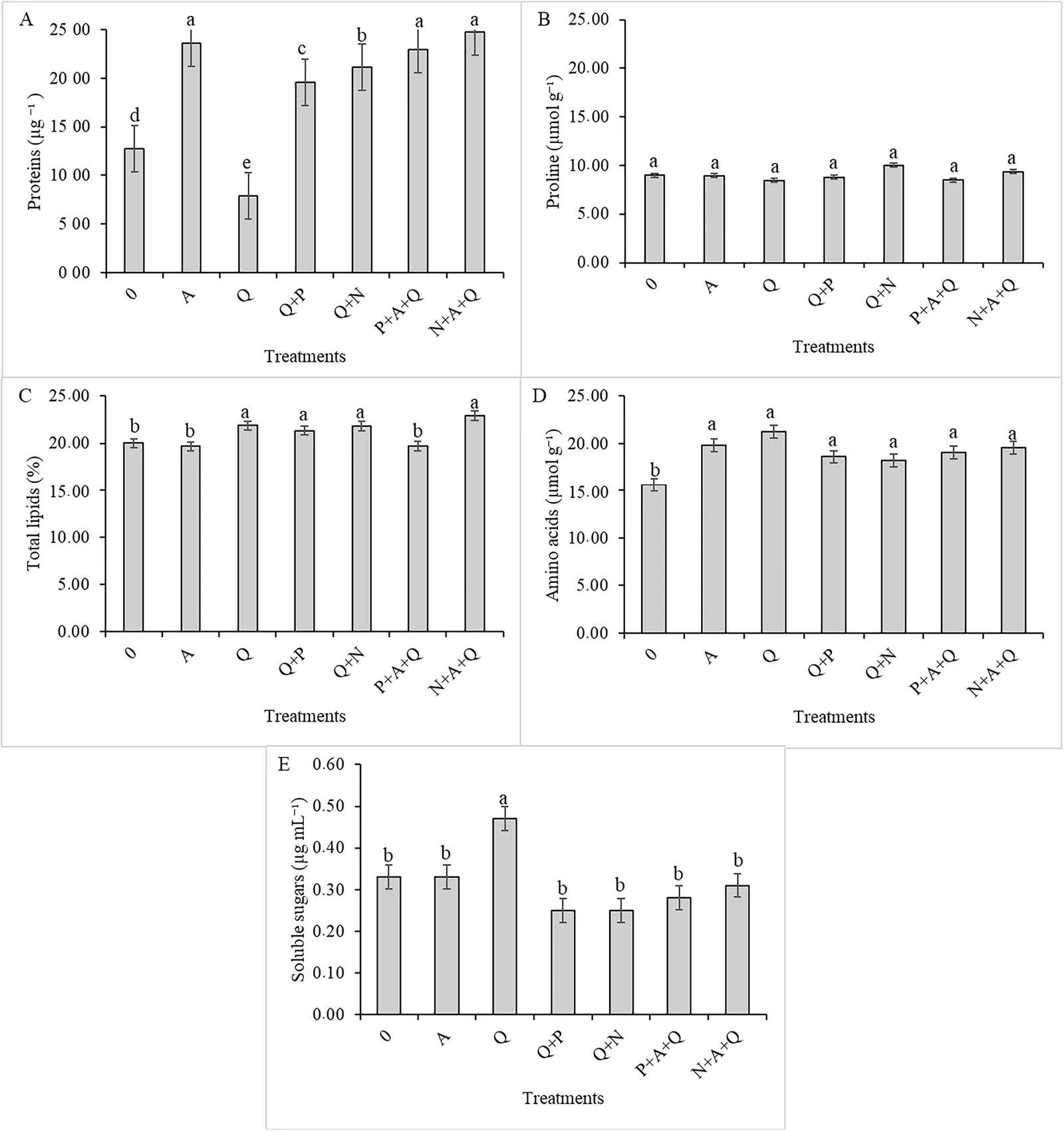
Content of protein (A), proline (B), lipids (C), amino acids (D), and total soluble sugars (E) in soybeans subjected to different seed treatments. Different letters indicate that the means belong to different groups based on the Scott-Knott test at a 5% significance level. Source: Prepared by the author.

The (Q + N) combination yielded intermediate protein levels, while the (Q + P) treatment yielded lower protein levels than the (Q + N) treatment (Figure 5A). The control treatment (0), without the application of inputs, showed moderate protein content, surpassing only the treatment with the chemical treatment alone (Q), which exhibited the lowest value (Figure 5A). It is worth noting that the results shown in (Figure 5A) indicate that *Azospirillum spp*., both when used alone and in combination with pyridoxine, nicotinamide, and the chemical input, led to more significant improvements in seed protein content, whereas the use of the chemical input alone resulted in the lowest physiological performance. These findings reinforce the positive effect of biological combinations and biostimulants on the biochemical composition of seeds (Figure 5A).

The proline concentration in soybeans did not show significant differences among the treatments evaluated. Both the control and the single applications (A) and (Q) resulted in statistically similar values. Similarly, the (Q + P) and (Q + N) treatments did not cause a detectable change in proline content (Figure 5B). The more complex combinations, involving (Q + P + A) and (Q + N + A), also maintained proline levels equivalent to those of the other treatments. The consistency of the results indicates that none of the interventions, whether used alone or in combination, influenced the synthesis or accumulation of this osmoprotective amino acid under the experimental conditions analyzed (Figure 5B).

Lipid quantification revealed significant differences among the treatments. The control group had one of the lowest lipid contents, similar to treatment (A) and also to the combination (Q + P +A). These results indicate that these conditions did not promote an increase in the lipid fraction of soybeans (Figure 5C). In contrast, treatment (Q) alone or in combination with the compounds (Q + P) and (Q + N) resulted in higher lipid contents. The triple combination of (Q + N + A) also showed a significant increase in the observed lipid levels (Figure 5C).

With regard to amino acid content (Figure 5D), no significant difference was observed among the treatments that received the products, which showed statistically similar values. In contrast, the control treatment (0) had significantly lower amino acid content; thus, the main effect observed indicates that the application of seed treatments increased amino acid content compared to the untreated control, regardless of the combination used.

With regard to total soluble sugars (Figure 5E), a significant effect of the treatments was observed, particularly for treatment Q, which had the highest content and differed statistically from all others (*p* ≤ 0.05). The treatments 0, A, Q + P, Q + N, P + A + Q, and N + A + Q did not differ from one another, exhibiting lower soluble sugar contents. This result indicates that the increase in total soluble sugars was specific to treatment Q; no additive or synergistic effect was observed when it was combined with other products.

The heat map of Pearson correlations revealed consistent relationships between seed vigor, early growth, physiological attributes, and soybean productivity components (Figure 6), allowing for the identification of positive and negative association patterns, as well as the strength and statistical significance of these relationships.

**Figure 6:**
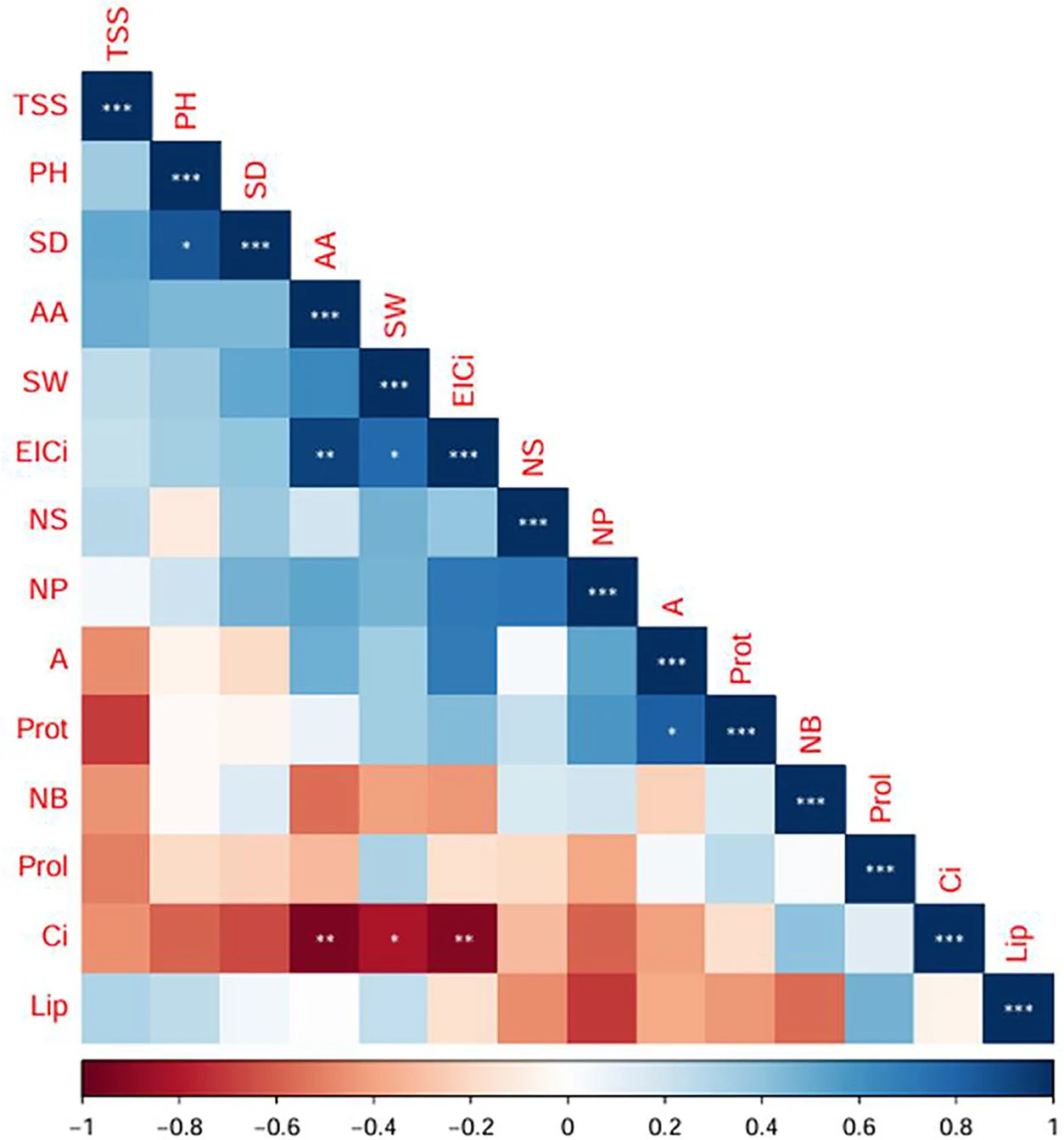
Heat map of Pearson correlations (correlation matrix) between seed vigor and the attributes of early growth, development, and productivity of the soybean crop, as a function of the application of chemical, biological, and vitamin products, used alone and in different combinations. Symbols *, **, and *** indicate significant correlations at *p* < 0.05, *p* < 0.01, and *p* < 0.001, respectively. Legend: number of pods per plant (NP), number of seeds (NS), seedling dry weight (SW), stem diameter (SD), plant height (PH), number of branches (NB), intracellular CO_2_ concentration (Ci), net photosynthesis (A), carboxylation efficiency (EICI), protein (Prot), lipids (Lip), proline (Prol), amino acids (AA), total soluble sugars (TSS). Source: Prepared by the author.

In general, a positive and significant correlation was observed between the variables associated with vegetative growth and productivity, particularly plant height (PH), stem diameter (SD), seedling dry weight (SW), number of seeds (NS), and number of pods (NP), indicating that increases in vigor and early development are reflected in improved crop yield. The correlations between PH and SD, as well as between SW and SD, were high in magnitude and statistically significant (*p* < 0.001), demonstrating a strong interdependence between these morphological attributes (Figure 6).

The physiological variables net photosynthesis (A) and intracellular CO_2_ concentration (Ci) showed a significant positive correlation with productivity components, particularly NS and NP, suggesting that higher photosynthetic efficiency is associated with increased plant productivity potential. In contrast, Ci showed a significant negative correlation with growth and vigor variables such as SW, SD, and PH, indicating that lower Ci values are associated with greater carbon assimilation efficiency and better vegetative performance (Figure 6).

Among the biochemical variables, total soluble sugars (TSS) showed a significant positive correlation with pH, SD, SW, and EICI, highlighting the importance of carbohydrate metabolism in supporting early growth and photosynthetic efficiency. Amino acids (AA) also showed a positive association with growth variables, albeit to a lesser extent, indicating a complementary role in plant metabolism (Figure 6).

On the other hand, proline (Prol) showed a significant negative correlation with growth and productivity variables, such as PH, SD, SW, NS, and NP, suggesting that higher levels of this Osmolite are associated with conditions of greater physiological stress, which negatively affects the plants’ agronomic performance. A similar trend was observed for protein (Prot), which showed a negative correlation with growth and physiological variables, albeit to a lesser extent (Figure 6). Lipids (Lip) showed a positive correlation with Ci and Prol, and a negative correlation with growth and productivity variables, indicating that their accumulation may be related to metabolic adjustments under conditions of lower physiological efficiency (Figure 6).

Overall, the correlation matrix shows that seed treatments with chemical, biological, and vitamin products had different effects on the physiological, morphological, and biochemical attributes of soybeans, with greater vigor and physiological efficiency being associated with vegetative growth and productivity, while the accumulation of stress-related compounds, such as proline, is inversely associated with these parameters (Figure 6). As Pearson correlation describes association rather than cause and effect, these relationships should be interpreted as evidence of coordinated physiological responses among traits, not as proof that any single variable drove the changes observed in another.

Principal component analysis showed that the first two canonical axes together explained 78.9% of the total variability in the data, with 56.5% attributed to Can1 and 22.4% to Can2 (Figure 7), indicating an adequate representation of the relationships between treatments and the variables evaluated. Can1 was strongly associated with variables related to physiological and productive performance, notably plant height (PH), total soluble sugars (TSS), stem diameter (SD), intracellular CO_2_ concentration (Ci), and number of seeds (NS), which exhibited the highest positive loadings on this axis. These results indicate that treatments positioned in the positive quadrant of Can1 are related to better physiological performance, greater vegetative growth, and higher productive potential (Figure 7).

**Figure 7:**
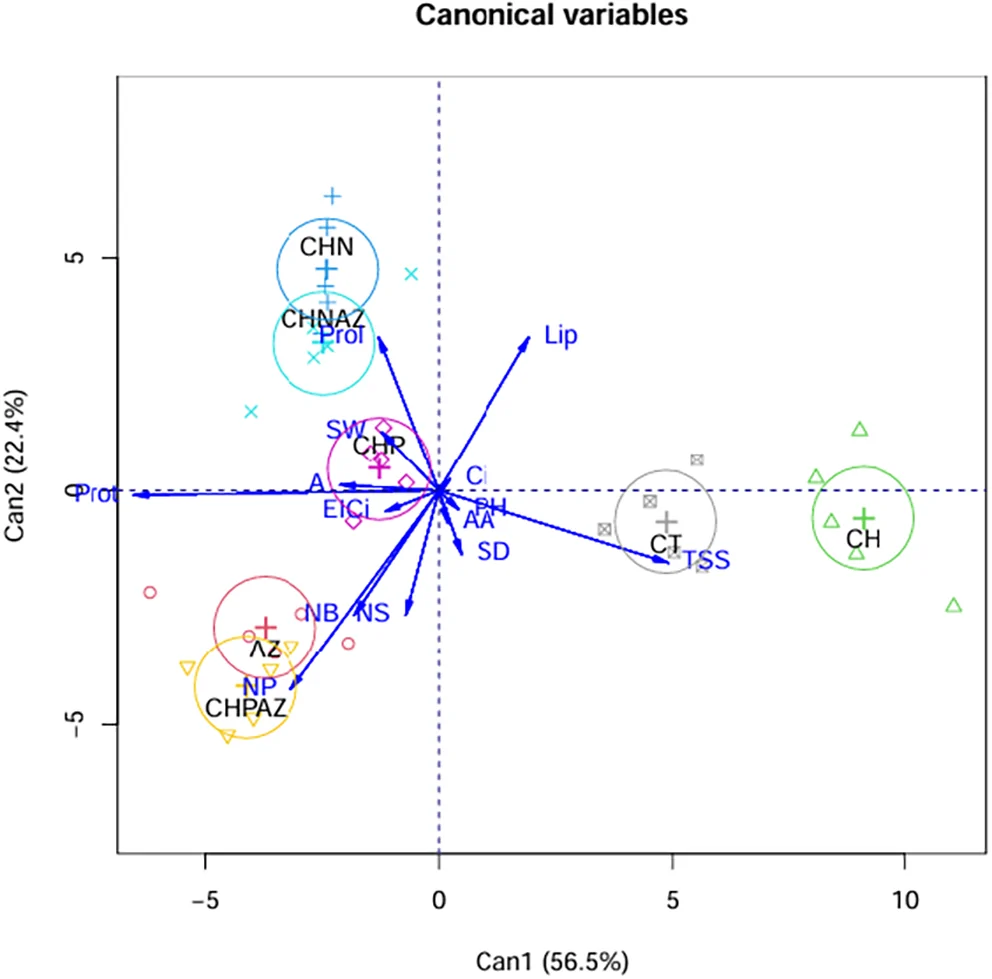
Principal component analysis of soybean seed treatments with chemical, biological, and vitamin products, applied individually and in combinations, in relation to the variables of vigor, initial growth, vegetative development, and crop productivity, as well as seed yield. Abbreviations as defined in Figure 6. Source: Prepared by the author.

In contrast, variables such as number of pods (NP), number of branches (NB), and seedling dry weight (SW) clustered in the negative quadrant of Can1, suggesting that certain treatments primarily favored traits related to initial establishment and plant architecture, but were less strongly associated with physiological variables and final yield (Figure 7).

Can2, in turn, was primarily influenced by biochemical variables, notably protein (Prot), lipids (Lip), proline (Prol), and amino acids (AA). The distribution of these variables along the vertical axis indicates that the treatments differed in terms of metabolic responses and the accumulation of organic compounds in seeds and plants, reflecting possible mechanisms of stress tolerance and metabolic efficiency induced by the products applied in seed treatment (Figure 7).

It was observed that chemical-based treatments, whether used alone or in combination, showed a stronger association with physiological and productive variables, such as pH, SD, Ci, and TSS, and were predominantly located in the positive quadrant of the Can1 plot. In contrast, treatments involving biological products and vitamins, either alone or in combination, showed a closer relationship with biochemical variables such as Prot, Lip, Prol, and AA, indicating a more pronounced influence on the metabolism and biochemical composition of seeds and plants (Figure 7).

Overall, the multivariate analysis showed that the different seed treatments produced distinct and complementary responses, making it possible to distinguish between treatment groups that had a greater effect on vigor and early growth, others on vegetative and physiological development, and those more closely related to seed yield and biochemical quality, underscoring the complexity of interactions between chemical, biological, and vitamin-based products in soybean seed treatment (Figure 7).

Figure 8 summarizes the positive impact of the findings, which demonstrate that seed treatments combining chemical products, *Azospirillum spp*., and vitamins B6 and B3 can modulate physiological responses, growth, yield components, and the biochemical composition of soybean seeds. The effects varied by treatment, notably highlighting the contribution of *Azospirillum spp*. to yield-related traits and protein accumulation, and that of treatments containing chemical products to growth and lipid composition. These results reinforce the potential of multi-input presowing management as a strategy to modulate crop performance and seed quality, although field studies are required to confirm its applicability.

**Figure 8:**
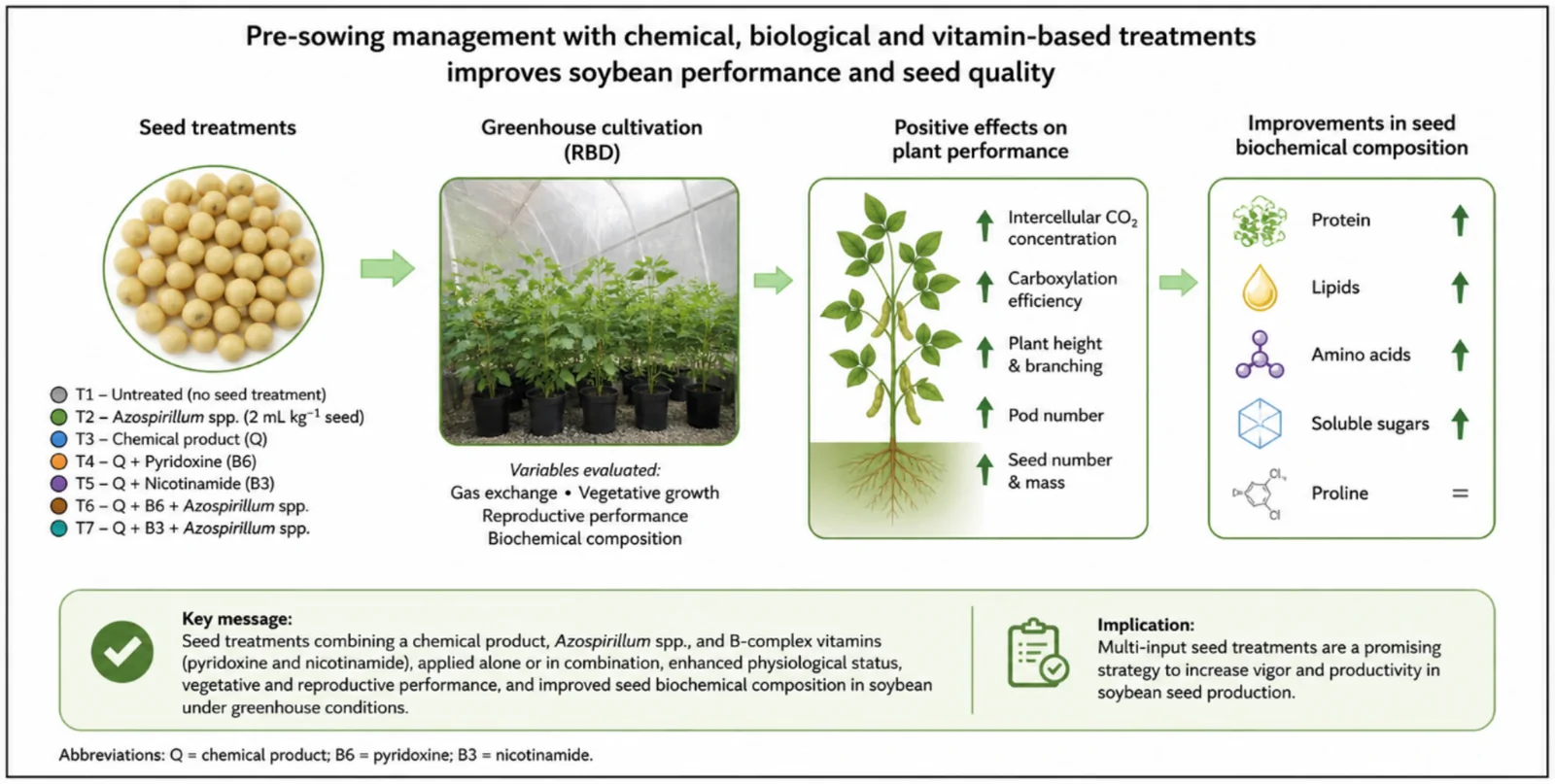
Schematic representation of the effects of chemical, biological, and vitamin-based seed treatments on the physiological performance, vegetative growth, productivity, and biochemical composition of soybean seeds under greenhouse conditions. The illustration was prepared by the authors and subsequently reviewed for scientific accuracy.

## Discussion

Several studies have shown that the application of chemical, biological, and vitamin-based inputs in seed treatment contributes to improved plant physiological performance, promoting greater uniformity in emergence and increased seedling vigor key factors for the proper initial establishment of the crop and, consequently, for increased agricultural productivity.^14,25^ These effects are directly reflected in the physiological processes associated with gas exchange and photosynthetic metabolism, which were evaluated in the present study.

For the transpiration rate (E), no significant differences were observed among the treatments (Figure 2C), indicating that the application of the inputs did not significantly alter water vapor flux through the stomata. This behavior suggests that plants maintained water balance regardless of the treatment applied, which is consistent with the concept of physiological stability under adequate water availability conditions.^26^ The absence of variation in this parameter allows us to infer that the responses observed in the other physiological variables did not result from changes in transpiration.

In contrast, the intercellular CO_2_ concentration (C_i_) showed significant variation among treatments (Figure 2D), with higher values in untreated seeds and in the QP treatment, while the others exhibited lower values. The reduction in C_i_ may be associated with both an increase in the rate of photosynthetic fixation and greater stomatal restriction of CO_2_ entry. On the other hand, treatments that maintained higher C_i_ values indicate less stomatal limitation, ensuring greater availability of CO_2_ in the mesophyll for carboxylation.^27^

This difference indicates that the treatments had distinct effects on the balance between carbon diffusion and assimilation. These results become particularly relevant when considered under heat stress conditions, since high temperatures can compromise plant growth and development, reduce the photosynthetic rate, and shorten the crop cycle.^26,28^ In the present study, a lower net CO_2_ assimilation rate was observed in plants grown from seeds not treated with pyridoxine, suggesting that this compound may have mitigated the effects of stress on the soybean photosynthetic apparatus. This finding is supported,^29^ who reported increased antioxidant enzyme activity in cucumber plants treated with pyridoxine via the seed, contributing to greater stress tolerance.

The interaction between stomatal conductance, internal CO_2_ concentration, and photosynthetic performance supports this interpretation. According,^30^ an increase in C_i_ accompanied by higher stomatal conductance indicates reduced stomatal limitation and greater photosynthetic efficiency. In contrast, under stress conditions, elevated C_i_ may be associated with nonstomatal limitations, such as reduced Rubisco activity, compromising carbon assimilation. Thus, the differences observed among the treatments suggest distinct physiological regulatory mechanisms in response to the imposed environmental conditions.

Despite these physiological variations, water use efficiency (WUE) did not show significant differences among the treatments, with mean values close to 6.0 μmol CO_2_ mmol^–1^ H_2_O (Figure 2E). This result indicates that the relationship between CO_2_ assimilation and transpiration was maintained, suggesting that the treatments did not compromise the crop’s water use efficiency. According,^31^ the stability of WUE reflects the absence of significant changes in stomatal conductance capable of affecting water use efficiency by plants, even in the face of internal physiological variations.

With regard to carboxylation efficiency, recent studies suggest that its reduction may be related to lower Rubisco activity, influenced both by competition with its oxygenase activity and by sensitivity to intracellular conditions.^32^ Furthermore, the regulation of this enzyme by stroma metabolites, such as RuBP levels and magnesium ions, may limit its efficiency under certain physiological conditions.^33^ In this context, the results obtained in this study (Figure 3A, B, C, and D) are consistent with the findings,^34^ who demonstrated that pyridoxine has a protective effect on plant cells, promoting greater resistance to biotic stresses.

Consistently^35^ reported that the application of pyridoxine significantly promoted plant development and resulted in increases in the yield and physiological quality of sesame seeds. Similar results were observed in different crops, such as corn,^12^ wheat,^34^ cucumber,^29^ tomato,^13^ and sesame,^36^ reinforcing the potential of this compound as a biostimulant with broad agricultural applicability.

It is important to clarify the biological meaning of the vegetative and reproductive traits discussed above, since they reflect distinct physiological processes rather than a single generic outcome. Plant height results primarily from internodal cell elongation and is a widely used, integrative indicator of vegetative vigor, since it depends on hormonal status (particularly auxins and gibberellins) and on the photoassimilate supply available for stem growth. Stem diameter and the number of branches, in turn, are structural traits associated with dry-matter accumulation and canopy architecture, determining the number of nodes available for flowering and, consequently, the plant’s reproductive capacity. Pod number is a direct component of reproductive yield, established during the reproductive stages (R1–R6) as a function of the successful conversion of flowers into pods, a process highly sensitive to the plant’s carbon and nitrogen status at flowering. Because these traits are governed by different physiological mechanisms, the seed treatments evaluated here did not necessarily affect them in the same way or through the same inputs: as shown in Figures 3 and 4, height gains were more consistently associated with the chemical-containing treatments (T3, T6, and T7), whereas pod number benefited more specifically from the Azospirillum-based treatments (T2 and T6), indicating that vegetative growth and reproductive output responded to at least partially distinct physiological stimuli.

In the present study, the growth and yield gains shown in Figures 3 and 4 are consistent with the adaptive mechanisms reported elsewhere for pyridoxine, such as increased antioxidant activity and improved root development; however, since antioxidant enzymes, reactive oxygen species, and root traits were not measured here, this interpretation should be regarded as a hypothesis for future testing rather than a demonstrated mechanism. Furthermore^17^ demonstrated that vitamin B6 stimulates the production of endogenous auxins and positively influences chlorophyll and carotenoid levels, reinforcing its role in modulating metabolic processes essential for plant growth, in agreement with the results observed in soybean crops.

In addition, the application of nicotinamide also had a positive effect on soybean growth and productivity, as shown in Figures 3 and 4, corroborating the findings.^37^ Nicotinamide has been associated with promoting vegetative growth, the accumulation of reserves, and increased grain yield, even under adverse conditions such as water deficit and salinity.^15^ Similar results have been reported in barley,^38^ corn,^39^ and oats,^40^ reinforcing its potential as an agent for mitigating environmental stresses.

With regard to biological treatments, seed inoculation with *Azospirillum spp*. had a positive effect on the physiological and yield parameters of soybeans (Figures 3 and 4), corroborating previous studies on corn and soybeans.^19,41^ The benefits associated with this microorganism stem mainly from the synthesis of phytohormones and improved stomatal conductance, factors directly related to photosynthetic efficiency and plant growth.^42,12^

Chemical seed treatments also had positive effects on the early development of soybeans (Figure 3A,B, and 3B,C), corroborating the findings,^43,44^ who reported stable or improved physiological performance in treated seeds. Additional studies demonstrate that the combination of chemical and biological treatments can result in significant productivity gains, as observed,^45^ who recorded higher yields in combined treatments compared to the control.

As shown in Figure 4A, B, and C, the positive effect of the treatments during the reproductive phase was a key factor in increasing seed yield in this study. The isolated or combined application of bioactive compounds resulted in consistent responses, even under adverse environmental conditions, such as high temperatures, which can compromise flower and pod formation.^26^ These results corroborate studies on tomato^13^ and sesame,^35,36^ reinforcing the potential of the evaluated treatments to enhance the physiological and productive performance of soybeans.

It is worth noting that pyridoxine acts as a cofactor that contributes to carbon and nitrogen metabolism, while nicotinamide plays a direct role in redox processes and the maintenance of cellular energy balance.^46^ The interaction observed (Figure 5C) in the Q + N + A treatment suggests a synergistic effect between nicotinamide and *Azospirillum spp*., indicating that these biological agents act as growth promoters and can enhance nutrient uptake, improve redox status, as well as increase plant metabolic efficiency and promote carbon allocation for lipid synthesis.^9,8^

Before discussing the treatment-related changes in seed biochemical composition (Figure 5), it is useful to briefly define the compounds evaluated and their physiological roles in seed and seedling development. Storage proteins are nitrogen-rich macromolecules accumulated in the cotyledons during grain filling; besides representing a nutritional reserve, they are hydrolyzed into amino acids during germination and are precursors of the enzymes required for early seedling metabolism. Lipids constitute the main energy reserve in oilseed species such as soybean and are mobilized through β-oxidation to sustain the respiratory demand of the embryonic axis before the seedling becomes photosynthetically autotrophous. Free amino acids act both as direct building blocks for de novo protein synthesis during germination and as signaling and osmoregulatory metabolites, while total soluble sugars represent the most readily available energy source and osmotic regulator supporting the initial metabolic reactivation that occurs during imbibition. Because these four compounds differ chemically and serve different physiological functions, they are not expected to respond uniformly to seed treatment, and the results below are therefore discussed separately for each compound rather than as a single, generic biochemical effect.

Significant variations were observed among the treatments for protein content (Figure 5A). The control and the isolated application of the chemical input (Q) showed the lowest values, while inoculation with *Azospirillum spp*. promoted a significant increase, highlighting the potential of these rhizobacteria to stimulate metabolic pathways associated with nitrogen assimilation and metabolism.^19^ The combined treatments (Q + P) and (Q + N) yielded even more pronounced increases in protein content, indicating that these vitamins may act as metabolic cofactors, promoting the intensification of nitrogenous compound synthesis (Figures 5, 6, and 7). The Q + P + A and Q + N + A combinations maintained the highest protein contents, suggesting a synergistic effect between the biological product and the vitamin regulators in the biosynthesis and accumulation of storage proteins (Figures 5, 6, and 7).

It is worth noting that the variations observed in protein content may also be related to the genetic characteristics of the cultivars evaluated (Figures 5, 6, and 7). As described,^47^ oil and protein contents in soybean seeds have a defined genetic basis; however, they are strongly influenced by environmental factors, especially during the grain-filling period, a critical phase for the synthesis, accumulation, and deposition of reserves. Furthermore, differences in protein accumulation among treatments may result from the interaction between genotype, environment, and management, including the application of the tested products.^48^ This interaction is dynamic and can be modulated by agrotechnological practices, which can alter the magnitude of the seeds’ physiological response.^48,50^ Inoculation with *Azospirillum spp*. had a positive effect on protein content but did not influence proline or lipid levels, suggesting an effect more specifically directed at nitrogen metabolism.^49^ These results reinforce the potential of the integrated use of microbial biostimulants and vitamin regulators, combined with chemical inputs, as a strategy to improve the biochemical quality of soybean seeds.^50,51^

On the other hand, proline (Figure 5B) remained stable across treatments, indicating the absence of osmotic or oxidative stress during seed conditioning. Under the conditions of this study, the treatments likely acted as beneficial physiological stimuli rather than stressors, which explains the lack of accumulation of this osmoprotective amino acid. According,^52^ proline acts as an important modulator of physiological responses associated with tolerance to abiotic stresses, contributing to increased plant adaptability under adverse conditions.

This amino acid tends to accumulate in high concentrations when plants are subjected to stressors, performing functions related to osmotic adjustment, protein and membrane stabilization, and the mitigation of oxidative damage.^53^ Additionally, the increase in intracellular proline concentrations is directly related to the intensity and nature of the stress imposed on plants, reflecting a dynamic biochemical mechanism of response and adaptation.^54^

The increase in lipid content observed (Figure 5C) in the treatments containing inputs Q, Q + P, Q + N, and Q + N + A suggests that this combination promoted mechanisms associated with the biosynthesis or retention of lipid reserves. It is worth noting that lipids represent one of the main energy sources for germination and initial vigor, and changes in their content can directly affect the physiological quality of seeds.^55^

The findings suggest that pyridoxine and nicotinamide may act as cofactors in the synthesis of amino acids and lipids, while *Azospirillum spp*. contributes to the production of phytohormones and metabolic signals that promote nutrient uptake and reserve metabolism. Thus, the combined effect of these inputs suggests a synergistic pathway for improving seed biochemical quality. Furthermore, amino acids and soluble sugars (Figure 5D and E) may act as osmoprotectants under stress conditions, contributing to the maintenance of cellular metabolism.^56^

Taken together, the biochemical results reveal a clear divergence among treatments that should not be summarized as a single, generic “increase in proteins, lipids, amino acids and sugars,” since each compound responded to a different input. Protein accumulation was preferentially enhanced by the Azospirillum-based treatments (T2, T6, and T7), consistent with the role of this rhizobacterium in stimulating nitrogen assimilation. Lipid content, in contrast, increased mainly in the chemical-containing treatments not combined with the biological agent alone (T3, T4, T5, and T7), pointing to a carbon-allocation response more closely associated with the chemical seed treatment and its combination with vitamins. Amino acid content increased uniformly in every treated group relative to the untreated control, regardless of which input was applied, indicating a general, input-independent response to seed treatment itself rather than an effect specific to any single product. Total soluble sugars, on the other hand, increased exclusively in the chemical treatment applied alone (T3), with no additive or synergistic benefit when combined with the biological agent or the vitamins. This treatment-specific pattern indicates that the nitrogen-related reserve (protein) and the carbon-related reserves (lipids and soluble sugars) were mobilized through at least partially independent physiological pathways, and that only amino acid accumulation can be attributed to seed treatment in general rather than to a specific input class.

Increased protein and lipid content can promote greater vigor, viability, and early seedling performance, thereby facilitating crop establishment and contributing to greater yield stability (Figures 5, 6, and 7). The integration of microbial biostimulants, vitamin regulators, and chemical inputs aligns with current trends in sustainable management, reducing dependence on exclusively chemical treatments.^56^

As shown in Figure 5A,B,C,D, and E, the biochemical compounds in soybean seeds were altered depending on environmental conditions and the management strategies adopted, including sprays and treatments applied to seeds or foliage. Overall, the results demonstrate that the application of biostimulants has a significant influence on biochemical attributes associated with seed quality, corroborating the findings.^57^ According,^58,59^ who reported consistent relationships between environmental factors particularly temperature and the physiological and biochemical quality of seeds.

Regarding proline content (Figure 5B), no statistically significant differences were observed among the treatments evaluated, indicating that the biostimulants and the combinations tested had no effect on this metabolite. This finding suggests that the seeds did not exhibit metabolic responses typical of oxidative or osmotic stress as a result of the treatments applied. These results highlight that proline accumulation occurs predominantly as a defense mechanism under conditions of severe chemical or environmental stress, which was not observed under the experimental conditions of this study.

Although the results demonstrate the potential of using vitamins and bioactive compounds in seed treatment to improve the physiological and productive performance of soybeans, several limitations of this study should be considered when interpreting and extrapolating these findings. The experiment was conducted entirely under controlled greenhouse conditions in pots, using a single soybean cultivar (69IX69), a single growing season, and one geographic region; claims regarding field-scale productivity and sustainability should therefore be treated as preliminary until confirmed under open-field conditions across seasons, cultivars, and environments. No molecular analyses (e.g., gene expression, enzyme activity assays) were performed, so the physiological and biochemical mechanisms proposed in the Discussion remain hypotheses rather than demonstrated pathways. Likewise, no economic or cost-benefit evaluation of the tested seed treatment combinations was conducted, and no long-term field validation was included. In this regard, it is recommended that these substances be evaluated in different cultivars, as well as under abiotic stress conditions. In addition, industrial-scale research on seed treatment and studies on the interaction of these compounds with the rhizosphere microbiota can help establish the use of these strategies in the physiological management of seeds and in improving crop productivity.

## Conclusions

Seed treatments altered intercellular CO_2_ concentration and carboxylation efficiency, without significant effects on net photosynthesis, stomatal conductance, transpiration, or water use efficiency, and also had a positive impact on the vegetative, reproductive, and biochemical characteristics of soybeans. Notable increases were observed in plant height, number of pods, and seed number and mass, as well as changes in protein, lipid, amino acid, and soluble sugar contents. Overall, the combination of chemical, biological, and vitamin-based products demonstrated greater efficiency, resulting in improved physiological performance, growth, and crop productivity.

## Data Availability

Data will be made available on request.
